# A breath of fresh air: the role of oxygen carriers in plastic surgery

**DOI:** 10.3389/fbioe.2026.1938458

**Published:** 2026-09-02

**Authors:** Leon Guggenheim, Valeria Pruzzo, Francesca Bonomi, Ettore Limido, Andrea Weinzierl, Matthias W. Laschke, Yves Harder

**Affiliations:** 1 Institute for Clinical and Experimental Surgery, Saarland University, PharmaScienceHub (PSH), Homburg, Germany; 2 Department of Surgery, Ospedale Beata Vergine Mendrisio, Ente Ospedaliero Cantonale (EOC), Mendrisio, Switzerland; 3 Department of Plastic, Reconstructive, and Aesthetic Surgery, Ente Ospedaliero Cantonale (EOC), Lugano, Switzerland; 4 Department of Plastic Surgery and Hand Surgery, University Hospital Zurich, Zurich, Switzerland; 5 Department of Plastic, Reconstructive, and Aesthetic Surgery and Hand Surgery, Centre Hospitalier Universitaire Vaudois (CHUV), Lausanne, Switzerland; 6 Faculty of Biology and Medicine, University of Lausanne (UNIL), Lausanne, Switzerland

**Keywords:** burn care, ischemia, oxygen carrier, plastic surgery, tissue hypoxia, wound healing

## Abstract

Tissue hypoxia represents a major challenge in regenerative medicine because it may impair wound healing and contribute to tissue necrosis and eventually reconstructive failure. To overcome this problem, topically applied oxygen carriers have emerged as a promising strategy to enhance tissue oxygenation independently of vascular function. This narrative review summarizes the clinical evidence for the use of oxygen carriers in plastic, reconstructive and aesthetic surgery. Four principal categories were identified, including hemoglobin-, peroxide-, and microalgae-based oxygen carriers as well as physical oxygen carriers. Hemoglobin- and peroxide-based oxygen carriers have shown beneficial effects on wound healing. However, evidence from randomized controlled trials remains inconsistent and large-scale studies for other indications are limited. In an early clinical study, microalgae-seeded scaffolds enabled controlled *in situ* oxygen production and provided structural support for full-thickness skin defects. Physical oxygen carriers have demonstrated positive preliminary results in promoting re-epithelialization of donor sites of split-thickness skin grafts but otherwise remain insufficiently investigated. Taken together, oxygen carriers represent a biologically plausible strategy to prevent tissue hypoxia. Although current clinical evidence suggests potential benefits, the literature on oxygen carriers remains limited by small patient cohorts, heterogeneous methodologies, and retrospective study designs. Therefore, future research should prioritize randomized controlled trials with standardized outcome measures and direct comparisons with established treatment modalities.

## 1 Introduction

Hypoxia occurs when oxygen delivery into the tissue is insufficient to maintain cellular homeostasis. It is characterized by impaired mitochondrial function, leading to an anaerobic shift in cellular metabolism, resulting in reduced adenosine triphosphate production and lactic acid accumulation. If not controlled, these processes ultimately result in cell death and organ dysfunction (Mallat et al., 2022). Tissue hypoxia may result from several mechanisms, among which ischemia represents the predominant cause in surgical settings. Ischemia is defined as a restricted or absent blood flow, thereby impairing oxygen delivery into tissues (Mallat et al., 2022).

The susceptibility of tissues to acute and persistent ischemia varies according to their metabolic demand. Metabolically highly active tissues, such as the nervous system, are particularly vulnerable to hypoxic injury and sustain irreversible damage earlier than tissues like muscle or skin (Ames and Nesbett, 1983; Picard-Ami et al., 1990). In plastic, reconstructive, and aesthetic surgery, acute ischemia is frequently encountered, for instance in traumatic injuries with acutely damaged vessels or vascular complications after flap surgery. In such circumstances, the surgeon must balance tissue preservation against the risk of ischemia and select reconstructive measures accordingly. When this balance is disrupted, the resulting hypoxia may lead to impaired wound healing, necrosis, and subsequent complications (Hom and Ostrander, 2023).

In addition to acute ischemia, many tissues relevant to reconstructive surgery exist in a state of chronic hypoxia or critically insufficient tissue perfusion, as observed in chronic wounds or otherwise altered tissues, such as irradiated skin. Similar to acute ischemia, chronic ischemia is often associated with micro- and/or macrovascular dysfunction (Moreira and Marques, 2022). Conventional approaches for the improvement of tissue oxygenation, such as pharmacological vasodilation, may be insufficient or ineffective when vascular integrity is compromised or absent, highlighting the need for alternative methods of oxygen delivery (Mallat et al., 2022). Established interventions, such as hyperbaric or topical oxygen therapy, provide effective means of enhancing tissue oxygenation (Ortega et al., 2021). However, these approaches are associated with significant limitations, including the need for a complex infrastructure, anatomical restrictions, and potential adverse effects (Ortega et al., 2021).

**FIGURE 1:**
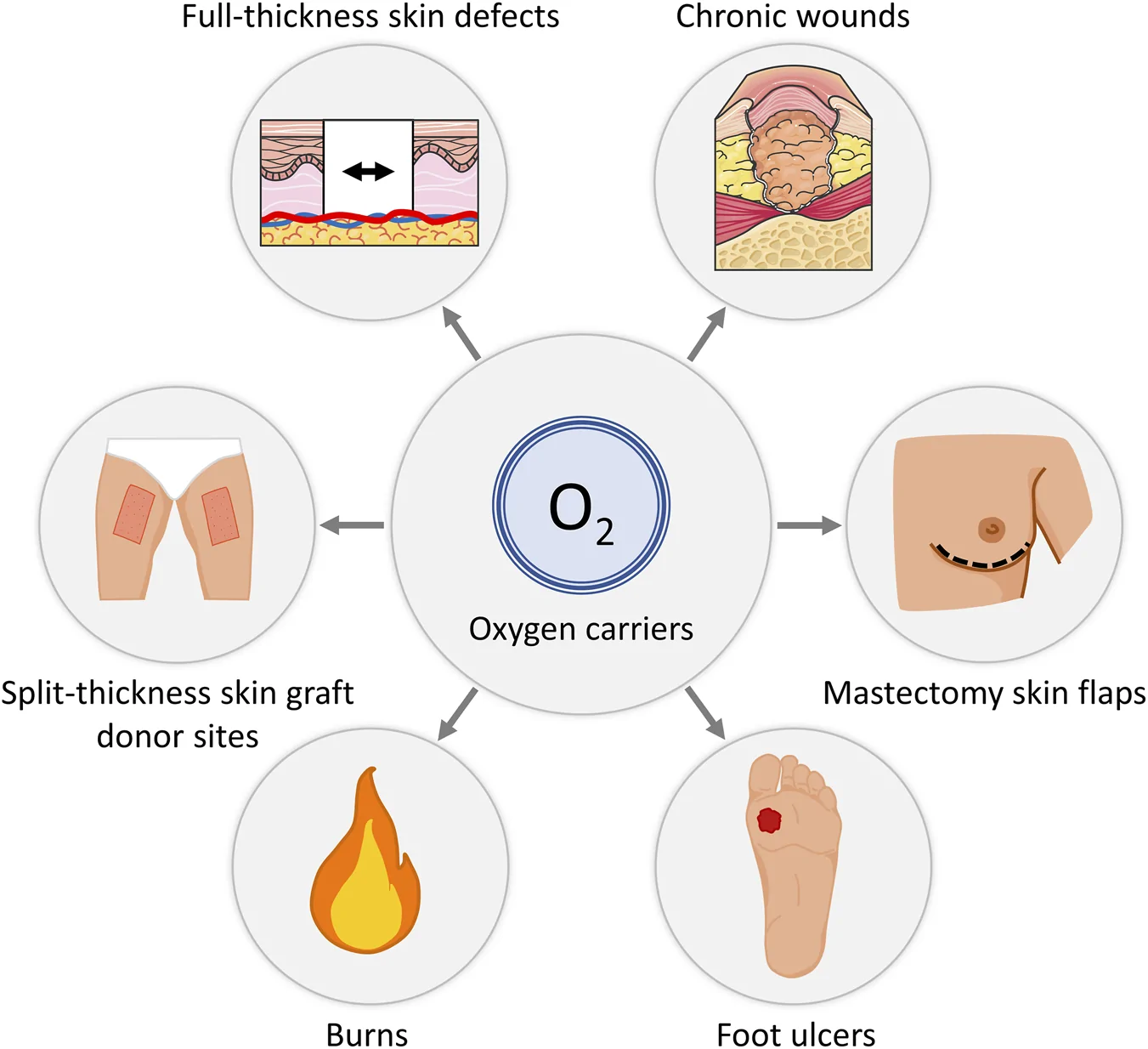
Potential clinical applications of oxygen carriers. The current literature provides evidence for several applications of oxygen carriers relevant to plastic, reconstructive, and aesthetic surgery. These include chronic wounds, mastectomy flap necrosis, foot ulcers, burn wounds, split-thickness skin graft donor sites, and full-thickness skin defects.

Oxygen carriers (OCs) represent a promising alternative strategy for topical oxygen delivery. OCs consist of varying substances capable of transporting or generating oxygen molecules. Initially developed as blood substitutes for managing hemorrhagic shock, they offer advantages, such as improved storability and independence from human donors. Although their clinical application as blood substitutes was discontinued, recent advances have renewed interest in their therapeutic potential and led to novel applications, such as organ preservation (Zhang et al., 2023; Barrou et al., 2025). In fact, topically applied OCs directly address the limitations of conventional approaches by enabling oxygen supply independent of a functional vasculature and, thus, must be viewed separately from blood substitutes. Accordingly, they are gaining increasing attention for the treatment of acute and chronic wounds, ischemic flaps, burn wounds, and split-thickness skin grafts (Figure 1). This narrative review provides a concise overview of topically applied OCs in plastic, reconstructive, and aesthetic surgery. By consolidating the currently available clinical evidence, we aim to highlight significant developments in the field and discuss potential future applications of these technologies.

## 2 Methods

**FIGURE 2:**
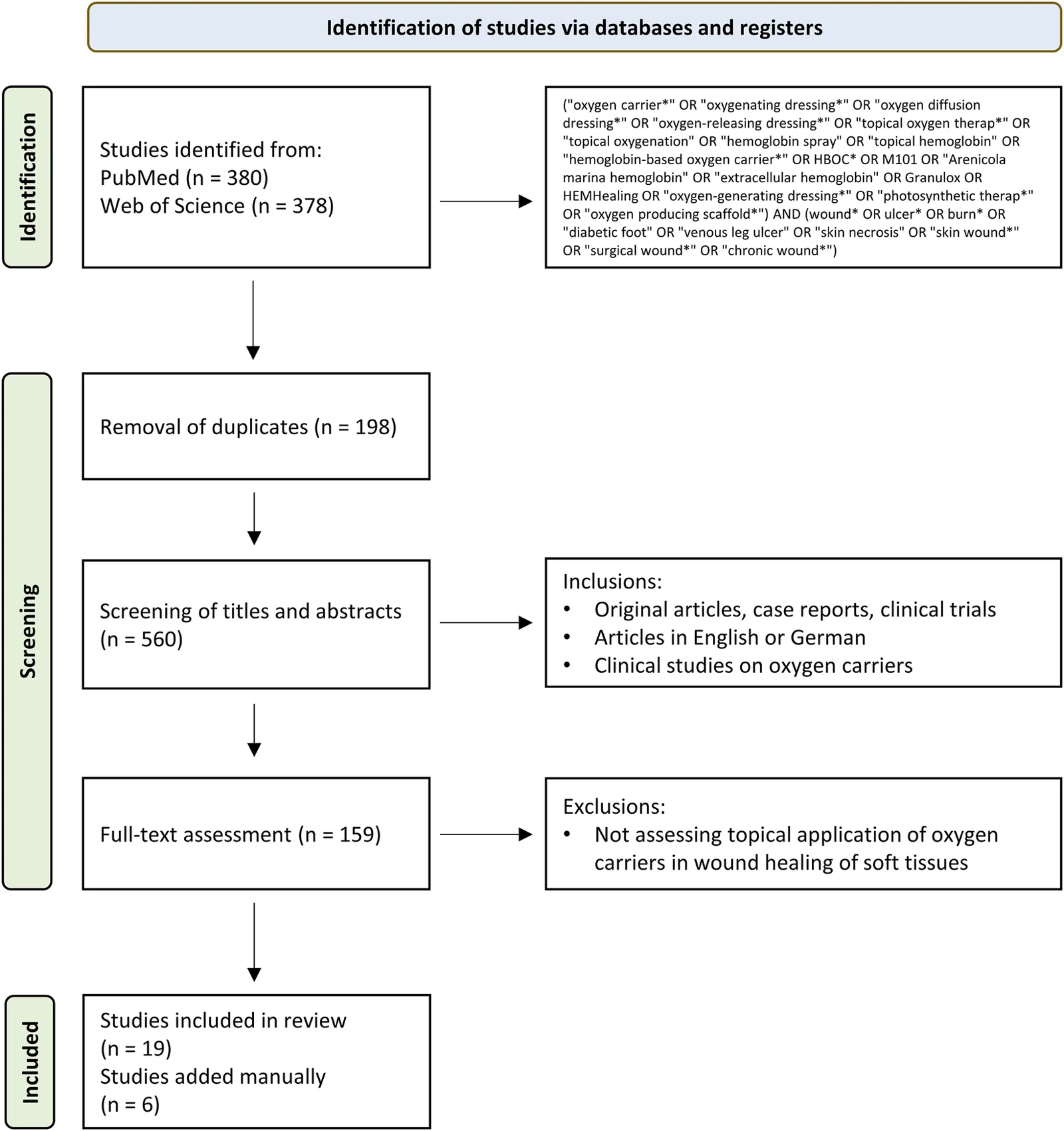
Study selection flowchart. Potential studies were identified through PubMed and Web of Science databases using a defined search string. After removal of duplicates, screening of titles and abstracts was performed according to the defined inclusion criteria followed by full-text assessment of included articles and exclusion of articles according to the exclusion criteria. Finally, 19 articles were included and 5 additional articles and one clinical trial were added manually.

A literature search was performed using PubMed/MEDLINE and Web of Science with the following search strategy (“oxygen carrier*” OR “oxygenating dressing*” OR “oxygen diffusion dressing*” OR “oxygen-releasing dressing*” OR “topical oxygen therap*” OR “topical oxygenation” OR “hemoglobin spray” OR “topical hemoglobin” OR “hemoglobin-based oxygen carrier*” OR HBOC* OR M101 OR “Arenicola marina hemoglobin” OR “extracellular hemoglobin” OR Granulox OR HEMHealing OR “oxygen-generating dressing*” OR “photosynthetic therap*” OR “oxygen producing scaffold*”) AND (wound* OR ulcer* OR burn* OR “diabetic foot” OR “venous leg ulcer” OR “skin necrosis” OR “skin wound*” OR “surgical wound*” OR “chronic wound*”). Only clinical studies published in English or German until the 1^st^ of June 2026 that evaluated the topical application of OCs in wound healing of soft tissues were assessed. Original articles, case reports, and clinical trials were included, whereas review articles, book chapters, and abstracts without complete data were excluded (Figure 2).

## 3 OC categories

**FIGURE 3:**
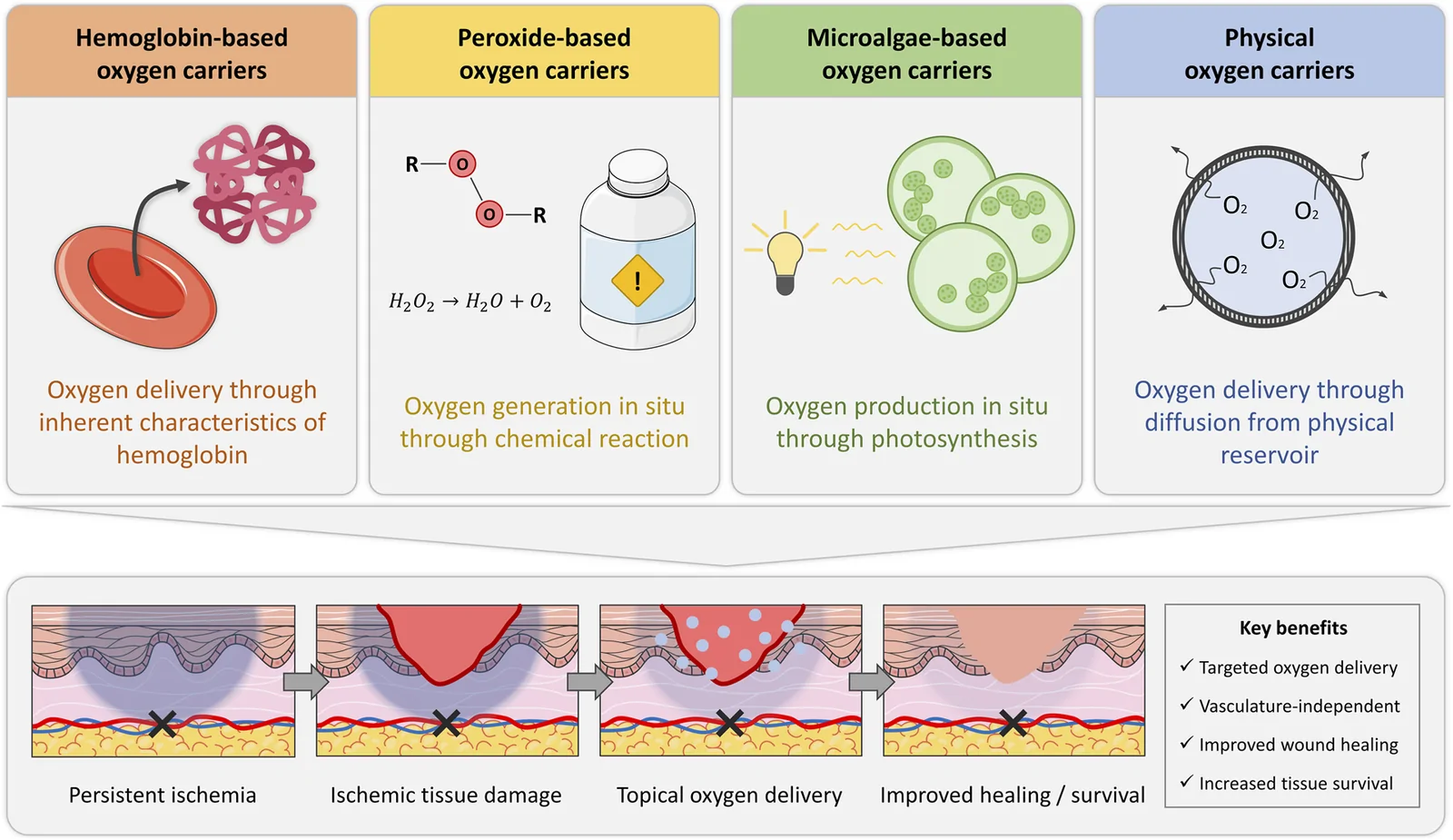
Categories and mechanisms of oxygen carriers. Four principal categories of oxygen carriers were identified in the current literature, including hemoglobin-, peroxide-, and microalgae-based, as well as physical oxygen carriers. Hemoglobin-based formulations can bind and release oxygen in a controlled manner depending on the demands of the surrounding tissue. Peroxide-based methods utilize the decomposition of peroxide bonds *in situ* to generate oxygen molecules. Microalgae-seeded scaffolds integrate photosynthetically active microalgae that generate oxygen in response to light exposure. Physical oxygen carriers deliver oxygen through diffusion from a preloaded reservoir to the underlying wound tissue. Through these mechanisms, oxygen carriers can oxygenate ischemic tissues independent of functional vasculature and, thus, promote wound healing and tissue survival.

Several categories of OCs have been described in the literature. However, only few have been translated to clinical practice until now, i.e., hemoglobin-based, peroxide-based, microalgae-based, and physical OCs (Table 1; Figure 3).

**TABLE 1:** Characteristics of oxygen carriers in plastic, reconstructive, and aesthetic surgery, as included in the present review.

Category	Oxygen delivery mechanism	Control of oxygen release	Duration of oxygenation	Dependence on external sources	ROS generation	Antimicrobial effects	Structural support
Hemoglobin-based	Heme group within hemoglobin binds and releases oxygen depending on the oxygen partial pressure	Regulated by oxygen gradient	Depending on degradation rate	Atmospheric oxygen	Hemoglobin may oxidize to methemoglobin – M101 as notable exception with intrinsic antioxidant activities	No	No
Peroxide-based	Chemical decomposition of peroxide group	Controllable through encapsulation, layered design, and addition of a catalyst	Depending on formulation and peroxide reserve	Generally independent but enzymatic systems may rely on atmospheric oxygen as a substrate	Presence or generation of H_2_O_2_ can be cytotoxic if not controlled – regulation through controlled release or enzymatic activity	Intrinsic activity from H_2_O_2_ and downstream products; additionally, through iodine in Oxyzyme®/Iodozyme®	No
Microalgae-based	Photosynthetic oxygen production under illumination	Externally modifiable by intensity and duration of illumination	Depending on microalgae viability	External light source	Minimal under controlled illumination – microalgae may have inherent antioxidant properties	Some microalgae may produce antimicrobial metabolites	Seeded onto dermal scaffold
Physical	Oxygen is physically entrapped or dissolved and released by diffusion along a concentration gradient	Limited control through material permeability	Depending on reservoir size	No	Minimal	No	No

ROS, reactive oxygen species.

**FIGURE 4:**
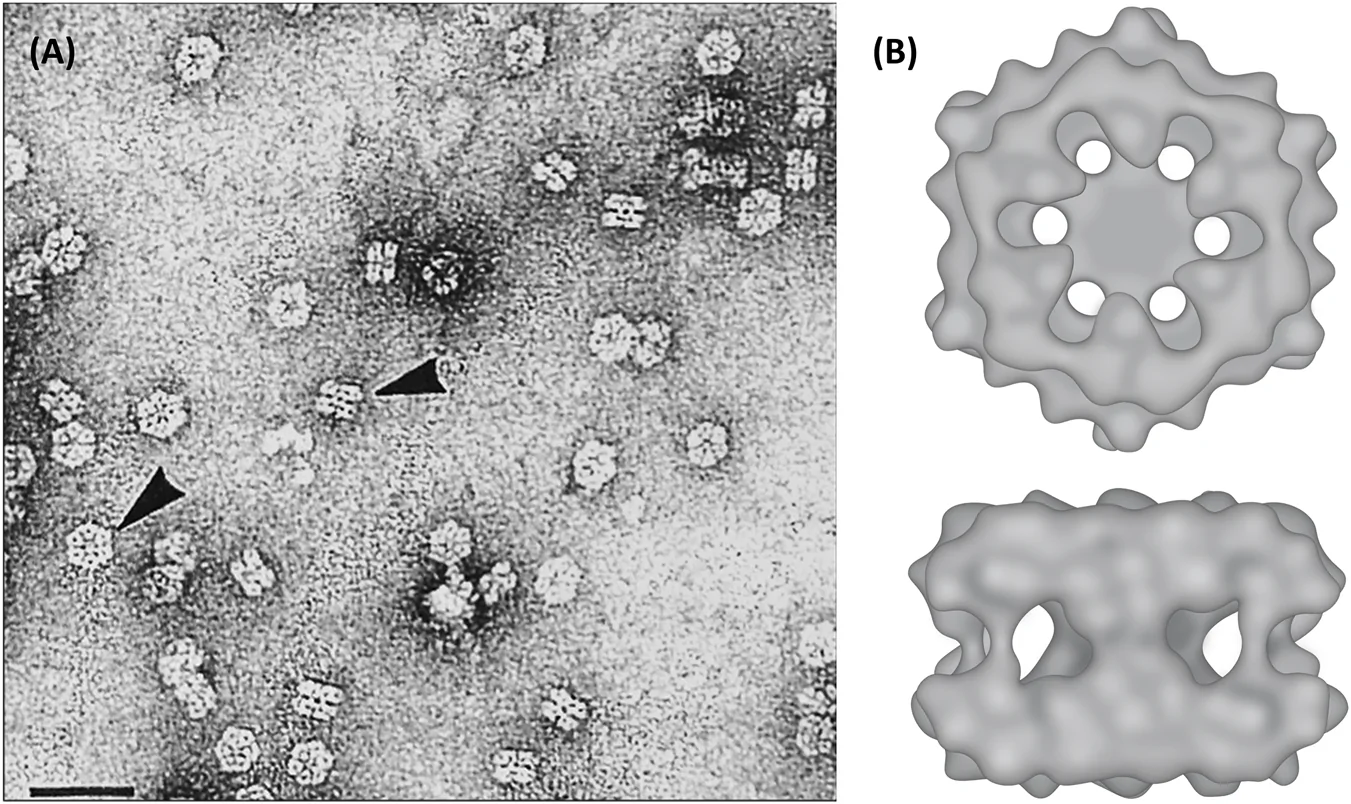
Structure of M101. **(A)** Electron micrograph of M101, negatively stained with 2% uranyl acetate showing the face and profile views (arrows). Scale bar: 60 nm. Reprinted with permission from Batool et al. (Batool et al., 2020), under the terms of the Creative Commons Attribution 4.0 International License. **(B)** Face and profile view of a three-dimensional reconstruction of M101.

Hemoglobin, the primary physiological oxygen carrier in vertebrates binds and releases oxygen in a controlled manner, depending on the demand of the surrounding tissue (Gell, 2018). Two types of hemoglobin are currently applied in patients, including porcine hemoglobin and the extracellular hemoglobin M101. Porcine hemoglobin contains four heme groups and exhibits properties comparable to those of human hemoglobin (Gell, 2018). M101 is extracted from the marine lugworm Arenicola marina and owing to its high oxygen-carrying capacity of up to 156 oxygen molecules per hemoglobin (Figure 4), M101 represents a promising candidate for topical oxygen delivery (Barrou et al., 2025). Hemoglobin-based OCs bind and release oxygen depending on the surrounding oxygen partial pressure and thus rely on the supply of atmospheric oxygen. While they may not directly produce reactive oxygen species, hemoglobin can be oxidized to methemoglobin which in turn generates reactive oxygen species and releases free iron (Rifkind et al., 2015). Notably, M101 is mentioned as an exception as it has demonstrated antioxidant properties due to an intrinsic superoxide dismutase-like activity (Rousselot et al., 2006).

An alternative approach to local oxygen delivery involves the use of peroxides that are characterized by two oxygen atoms connected through a covalent bond and interposed between varying atoms or molecules (Ma et al., 2021). These attached atoms and molecules determine certain properties of the peroxide and allow for further classification into liquid peroxides, including hydrogen peroxide, and solid peroxides. The latter include metal peroxides, such as calcium peroxide, that have gained increasing attention in wound healing applications (Ma et al., 2021; Bayraktar et al., 2024). The peroxide bond is inherently unstable and readily decomposes into molecular oxygen and a second product, such as water in the case of hydrogen peroxide or calcium oxide in the case of calcium peroxide (Ma et al., 2021). This decomposition can be exploited in wound dressings to provide a sustained local oxygen supply. In addition, peroxides exhibit antimicrobial activity, by generating reactive oxygen species and are therefore already in clinical use as disinfectants, making them particularly attractive for the treatment of infected wounds (Bayraktar et al., 2024). Though this generation of reactive oxygen species can be advantageous due to its antimicrobial activity, regulation through controlled release or enzymatic regulators is necessary to prevent cytotoxic effects on the wound tissue.

Photosynthetic biomaterials, specifically microalgae-based biomaterials, represent a promising strategy to address oxygen-deficient conditions across various clinical scenarios, including organ transplantation, tissue infarction, tumor therapy, and wound healing (Kang et al., 2026). The potential advantage of photosynthetic scaffolds is their ability to provide sustained oxygen delivery that can be regulated through *in situ* illumination without requiring frequent dressing changes. While the production of reactive oxygen species is inherent to the generation of oxygen molecules, certain microalgae may present inherent antioxidative properties and additionally may produce antimicrobial metabolites exerting an antimicrobial effect on the wound (Martínez-Ruiz et al., 2022).

Physical OCs allow the topical delivery of oxygen by physically entrapping oxygen without chemically binding it to a carrier molecule or generating it *de novo in situ*. The entrapped oxygen can then be released into the wound through diffusion, modifiable by differing permeabilities of the interfacing layer. Due to their design, physical OCs also do not rely on a continuous external oxygen supply.

## 4 Hemoglobin-based topical OCs

As previously described, two types of hemoglobin are currently in use for the treatment of patients with varying types of wounds. Granulox® (Mölnlycke Healthcare AB, Mölndal, Sweden) is a spray containing porcine hemoglobin that was developed for topical use in chronic wounds, such as diabetic foot ulcers, venous ulcers, and pressure sores. Unlike other clinically applied OCs, Granulox® has been extensively investigated in several case reports, case series, and randomized controlled trials (RCTs) examining its effects on wound healing Table 2.

**TABLE 2:** Overview of clinical studies on the application of oxygen carriers in plastic, reconstructive, and aesthetic surgery, as included in the present review.

Category	Oxygen carrier	Study type	Year	Number of patients	Target tissue	Regulatory status	Outcome	References
Hemoglobin-based	Porcine hemoglobin	Cohort	2016	15 control, 20 case	Diabetic foot ulcer	Postmarketing study	↑ Mean wound size reduction (95% vs. 63% after 26 weeks) ↓ Pain	(Hunt and Elg, 2016)
Hemoglobin-based	Porcine hemoglobin	Case series	2016	17	Diabetic foot ulcer	Postmarketing study	Mean wound size reduction of 54% after 4 weeks Positive rating by clinicians	(Haycocks et al., 2016)
Hemoglobin-based	Porcine hemoglobin	Case series	2016	13	Diabetic foot ulcer	Postmarketing study	Mean wound size reduction of 39% after 12 weeks	(Hunt et al., 2016)
Hemoglobin-based	Porcine hemoglobin	Case series	2023	12	Diabetes-related foot ulcer	Postmarketing study	Wound size reduction of 87%–100% (varying time points) Positive rating by clinicians	(Njokweni, 2023)
Hemoglobin-based	Porcine hemoglobin	Case report	2024	1	Diabetic foot ulcer	Postmarketing study	Successful wound healing after resistance to initial treatment	(Siafarikas et al., 2026)
Hemoglobin-based	Porcine hemoglobin	RCT	2021	14 control, 15 case	Foot ulcers of mixed etiology	Postmarketing study	No difference in wound size reduction	(Jonker et al., 2021)
Hemoglobin-based	Porcine hemoglobin	RCT	2013	31 control, 34 case	Venous ulcer	Postmarketing study	↑ Mean wound size reduction (53% vs. −21% after 13 weeks) ↑ Wound quality ↓ Pain	(Arenbergerova et al., 2013)
Hemoglobin-based	Porcine hemoglobin	Case report	2021	2	Venous ulcer	Postmarketing study	Successful wound healing as adjunct after interventions to treat underlying cause	(Loh et al., 2021)
Hemoglobin-based	Porcine hemoglobin	Mixed (RCT, case series, case reports)	2011	62	Chronic wounds of mixed etiology	Postmarketing study	RCT: ↑ wound healing (93% vs. 7% after 26 weeks); 39/42 (93%) with subsequent treatment of all patients after the study was closed Case series: Successful wound healing after 10 (8–12), 11 (4–20), and 17 (2-53) weeks	(Arenberger et al., 2011)
Hemoglobin-based	Porcine hemoglobin	Cohort	2018	95 control, 100 case	Sloughy chronic wounds of mixed etiology	Postmarketing study	↑ Wound healing (94% vs. 63% after 26 weeks) ↑ Early mean wound size reduction (52% vs. 11% after 1 week) ↑ Wound quality ↓ Pain	(Hunt et al., 2018)
Hemoglobin-based	Porcine hemoglobin	Cohort	2017	50 control, 50 case	Chronic wounds of mixed etiology	Postmarketing study	↑ Wound healing (90% vs. 38% after 26 weeks) ↓ Mean time to wound healing (7 weeks (3-22) vs. 11 weeks (3-25)) ↑ Wound quality ↓ Pain	(Hunt and Elg, 2017)
Hemoglobin-based	Porcine hemoglobin	Case-control	2026	31 control, 28 case	Burns (2nd degree)	Off-label use	Faster re-epithelialization Fewer dressing changes ↓ Late complications	(Kulice, 2026)
Hemoglobin-based	M101	Case report	2024	1	Burns (3rd degree)	Compassionate use	Successful re-epithelialization (fingertips)	(Awan et al., 2024)
Hemoglobin-based	M101	Case report	2025	1	Burns (2nd degree)	Compassionate use	Reduction of burn area to <2% after 6 weeks without skin grafts (trunk and abdomen)	(Lancien et al., 2025)
Hemoglobin-based	M101	Case report	2026	2	Mastectomy skin flap necrosis	Compassionate use	Aversion of full-thickness skin necrosis without surgical debridement	(Lhuaire et al., 2025)
Peroxide-based	Glucose oxidase	Case series	2007	4	Venous ulcer	Postmarketing study	Successful wound healing in all patients within 5 weeks	(Ivins et al., 2007)
Peroxide-based	Glucose oxidase	Case series	2009	100	Chronic wounds of mixed etiology	Postmarketing study	Mean wound size reduction of 35% after 6 weeks Positive rating by clinicians	(Davis et al., 2009)
Peroxide-based	Glucose oxidase	Case series	2010	45	Chronic wounds of mixed etiology	Postmarketing study	Mean wound size reduction of 20% after 6 weeks Positive rating by clinicians	(Wood et al., 2010)
Peroxide-based	Glucose oxidase	Case series	2011	11	Chronic wounds of mixed etiology	Postmarketing study	Mean wound size reduction of 52% after 6 weeks, 79% after 12 weeks, and 88% after 20 weeks Cost analysis suggests £23,036 savings over 52 weeks compared to standard treatment	(Lafferty et al., 2011)
Peroxide-based	Glucose oxidase	RCT	2014	53 control, 47 case	Chronic wound of vascular origin	Postmarketing study	No difference in wound size reduction Fewer dressing changes in case group	(Moffatt et al., 2014)
Peroxide-based	Sodium percarbonate and calcium peroxide	RCT	2026	69 control, 68 case	Skin graft donor site	Premarketing study	↓ Time until complete wound healing (11 days vs. 15 days) ↑ Wound healing rate in first 2 weeks ↓ Pain	(Tong et al., 2026)
Microalgae-based	Chlamydomonas reinhardtii	Phase 1 clinical trial	2021	8	Full-thickness skin defects	Experimental modification/early clinical investigation	Successful integration of the scaffold and overlying skin grafts No significant adverse events	(Obaíd et al., 2021)
Microalgae-based	Chlamydomonas reinhardtii	Case report	2022	1	Full-thickness skin defect	Experimental modification/early clinical investigation	Positive long-term outcome (17 months)	(Obaíd et al., 2022)
Physical	Gas reservoir	RCT	2014	17	Skin graft donor site	Postmarketing study	↓ Time until complete wound healing (9 days vs. 12 days) ↓ Pain	(Lairet et al., 2014)
Physical	Closed cell foam hydrogel (Polyacrylamide polymer)	RCT	2017	8	Abdominoplasty scar	Postmarketing study/discontinued product	No differences in any outcome	(Halyard Health, 2017)

RCT, randomized controlled trial; ↑, increased; ↓, decreased.

Diabetic foot ulcers represent an interesting target for OC-based therapies, because of their high prevalence and underlying microvascular dysfunction (Armstrong et al., 2023). A retrospective study by Hunt and Elg (Hunt and Elg, 2016) compared two cohorts of patients with non-healing diabetic foot ulcers, defined as <40% wound size reduction in 12 weeks, and a SINBAD (Ince et al., 2008) score of ≤2. A control group of 15 patients received standard wound care, including surgical debridement, standard wound dressings, and off-loading devices as required. Another group of 20 patients received identical standard treatment with the addition of Granulox® twice weekly, combined with dressing changes until wound closure was achieved. Wound size changes, complete wound healing, pain level, exudate quantity, and other factors were assessed over a 6-month period. Of interest, the study showed a significant 95% reduction in mean wound size in the Granulox® group, compared to 63% in the control group. Furthermore, the group treated with Granulox® showed an increased number of completely healed wounds and reduced pain scores (Hunt and Elg, 2016). Haycocks et al. (Haycocks et al., 2016) evaluated 17 patients with diabetic foot ulcers in a retrospective case series who underwent the same treatment regimen as described above, showing a mean wound size reduction of 54% after 4 weeks. Thirteen patients continued the treatment for an additional 8 weeks, resulting in a mean wound size reduction of 39% after a total treatment duration of 12 weeks (Hunt et al., 2016). Similar findings were reported in more recent retrospective studies, which show improved healing rates and positive clinician ratings, although not compared with a control group (Njokweni, 2023; Siafarikas et al., 2026). Conversely, the only prospective RCT in patients with foot ulcers conducted by Jonker et al. (Jonker et al., 2021) showed no improvement when comparing standard care alone, including surgical debridement, infection management, and offloading, with standard care plus Granulox® therapy. Patients with an ulcer present for at least 4 weeks and a healing rate of less than 50% were enrolled into the study, of which 14 patients were allocated to the control arm and 15 patients to the Granulox® arm. Throughout the study and after conclusion of treatment at 12 weeks, no significant differences in wound size could be observed between the groups (Jonker et al., 2021). The interpretation of these findings is complicated by the increased baseline wound chronicity in the Granulox® arm, which may influence the final outcome. However, this was not statistically analyzed in the study.

In addition to its use in diabetic foot ulcers, Granulox® was investigated for the treatment of other types of chronic wounds, such as venous and arterial ulcers and chronic wounds of mixed etiology. Arenbergerova et al. (Arenbergerova et al., 2013) conducted a prospective RCT investigating the use of Granulox® in venous leg ulcers. Initial enrollment included patients with venous leg ulcers present for over 8 weeks and a wound size of 2–50 cm^2^, resulting in 36 patients allocated to each of the control and Granulox® arm. Both groups received compression therapy, starting 2 weeks prior to trial initiation and were hospitalized during the first 2 weeks of the trial for daily dressing changes. The patients in the treatment group received Granulox® during dressing changes, whereas the control group received sham treatment with a saline spray. Of the 72 patients, 31 in the control arm and 34 in the Granulox® arm completed the study. The results show a significant 53% reduction in wound size after 13 weeks in the Granulox® group, compared with no change in wound size in the control group. Furthermore, the Granulox® group exhibited an improved wound quality, as indicated by reduced necrotic tissue, fibrin slough, and an increase in granulation tissue, as well as improvements in pain intensity compared to the control group (Arenbergerova et al., 2013). Similar outcomes were described in subsequent case reports of two patients with venous and mixed arterial-venous ulcers, respectively, who achieved successful healing after Granulox® therapy (Loh et al., 2021). Further studies investigated patient populations with chronic wounds of diverse etiologies, including diabetic and vascular causes as well as traumatic wounds or pressure ulcers. Arenberger et al. (Arenberger et al., 2011) reported cumulated retrospective data from several centers in Mexico, Germany, and the Czech Republic, evaluating a hemoglobin spray in chronic wound care. Although all centers followed a similar application protocol of the hemoglobin spray, substantial variability in adjacent standard wound care and study design can be observed. One center in Mexico conducted a prospective RCT comparing hemoglobin spray in 14 patients with a local standard treatment using a sterile petroleum jelly bandage in 14 control patients. After 6 months, the trial was discontinued due to distinct positive results. In the hemoglobin spray group, 13 of 14 patients healed successfully, compared with only one of 14 in the control group. Subsequently, all patients, including previously excluded patients, received the hemoglobin spray therapy and were enrolled in an observation study with complete healing in 39 out of 42 patients. Data from the other three centers consist mainly of individual cases, cumulating to a total of 20 patients. From these cases, all wounds healed completely with an average time until wound healing of 10 (8-12), 11 (4-20), and 17 weeks (2-53) at the three centers, respectively (Arenberger et al., 2011). A separate study by Hunt et al. (Hunt et al., 2018) investigated the use of Granulox® in chronic and sloughy wounds. In this cohort study, a group of 100 patients with any wound showing evidence of slough without requiring immediate hospitalization was trained on the at-home application of Granulox®, which they performed twice weekly until complete wound healing was achieved. This group was compared to 95 control patients receiving individual standard wound care that was not further specified. After a follow-up of 26 weeks, 94% of patients treated with Granulox® had healed completely compared to 63% in the control group. Furthermore, a more rapid wound size reduction and reduced pain during the first 8 weeks was observed in the Granulox® group (Hunt et al., 2018). However, significant differences in pain and slough coverage between the two groups with higher presence in the Granulox® group were observed, potentially affecting the outcome. In 2017, Hunt and Elg (Hunt and Elg, 2017) performed a similar cohort study with the same methodology, albeit on patients with chronic wounds that did not show fibrin coverage and failed to respond after 2–4 weeks of standard wound treatment. A group of 50 patients receiving Granulox® therapy was compared with 50 control patients, identified retrospectively, who received only standard treatment. This study showed similar results, with 90% of patients in the Granulox® group achieving complete wound healing after 26 weeks, compared with 38% in the control group. Furthermore, the time to complete wound healing was shorter in the Granulox® group with an average of 7 weeks compared to 11 weeks in the control group (Hunt and Elg, 2017). Taken together, the available evidence indicates a potential benefit for the local application of Granulox® as an adjunct in the treatment of chronic wounds. However, the interpretation of these findings is limited by methodological weaknesses and the retrospective design of most studies. Furthermore, substantial variation in patient populations, follow-up durations, and control treatments limit cross-study comparability.

Recently, a case-control study by Kulice (Kulice, 2026) investigated a novel application of Granulox® in pediatric patients with second-degree burns. In one group, 28 patients received Granulox® in addition to standard burn wound dressings at each dressing change compared with 31 patients in a control group who received standard wound care with paraffin-impregnated gauzes only. This study could show that treatment with Granulox® results in faster re-epithelialization, earlier hospital discharge, reduced need for dressing changes, and decreased incidence of long-term complications, including itching, inhomogeneous pigmentation, hypertrophic scarring, and epithelialization defects (Kulice, 2026). These findings suggest the potential applicability of Granulox® beyond chronic wound care.

In theory, burn wounds seem particularly well suited for topical oxygen therapy due to acutely impaired microvascular perfusion in superficial soft tissue layers, such as dermis and subcutaneous fat, and large wound areas. In this context, M101 has recently gained attention. Two recent case reports evaluated the use of HEMHealing® (HEMARINA S.A., Morlaix, France), a novel M101-containing hydrogel, in burn wounds. Awan et al. (Awan et al., 2024) reported complete re-epithelialization of third-degree fingertip burns in a 22-year-old woman treated with HEMHealing®. Lancien et al. (Lancien et al., 2025) reported the case of a 34-year-old man presenting with burns involving 85% of the total body surface area. Due to limited donor sites, HEMHealing® was applied to second-degree burns on the chest, abdomen, and back every other day for 47 days, with successful reduction of the burn area in these regions to less than 2% by week 6 (Lancien et al., 2025). More recently, Lhuaire et al. (Lhuaire et al., 2025) applied HEMHealing® in a different setting to treat acute persistent ischemia of the adipo-cutaneous skin flap following nipple-sparing mastectomy in two patients, with successful healing by 21 and 76 days without requiring additional debridement in the operating room.

Taken together, hemoglobin-based OCs have shown encouraging results across a variety of acute and chronic wounds. While numerous case series and cohort studies report improved outcomes, including reduced wound size and pain, evidence from RCTs remains inconsistent. M101 represents an emerging alternative due to its superior oxygen-carrying capacity with promising early clinical observations, particularly in burn care. Nonetheless, the current body of evidence is limited by heterogeneity in study designs and methodologies, underscoring the need for large-scale RCTs to better define the clinical efficacy and optimal application protocols.

## 5 Peroxide-based topical OCs

The first commercialized peroxide-based OC was Oxyzyme® (Crawford Healthcare Ltd., Loughborough, UK), which employed a layered enzymatic system to manage the unstable nature of peroxides and prevent excessive decomposition. The dressing contains glucose and potassium iodide in an inner layer and glucose oxidase in an outer layer. This allows for the continued production of hydrogen peroxide, which in turn reacts with potassium iodide to generate oxygen and iodine. A variation of this dressing marketed as Iodozyme® (Crawford Healthcare Ltd., Loughborough, UK), releases higher concentrations of iodine and is intended for use in infected wounds. Ivins et al. (Ivins et al., 2007) presented the first application of Oxyzyme® in a case series including 4 patients suffering from chronic venous ulcers. In this series, all wounds healed completely within 5 weeks, suggesting therapeutic potential, although limited by the small sample size and lack of untreated control patients. Similar findings were subsequently reported in a larger case series conducted by Davis et al. (Davis et al., 2009). The authors collected data from several centers across Europe, evaluating Oxyzyme® in superficial non-infected, chronic, hard-to-heal wounds defined as static or deteriorating during the previous 4 weeks under standard treatment, including compression bandaging, offloading, and other dressings. The dressing was applied after wound cleaning and was changed according to the state of the wound for 6 weeks or until complete healing, if occurring sooner. A total of 100 patients, of which 38 were excluded from the study prior to its conclusion due to infection, deterioration, pain, and/or other reasons, were analyzed and showed complete healing in 10%, improvement in 63%, unchanged wound conditions in 16%, and deterioration in 11%, with an overall mean wound area reduction of 35%. Subgroup analyses showed the largest reduction (54%) among patients with diabetic foot ulcers. However, the substantial dropout rate of 38% limits the interpretation of these results. Additionally, clinicians were asked for their subjective assessment of the dressing, resulting in 82% rating it as better or much better than standard dressings (Davis et al., 2009). Another case series by the same authors investigated the use of Iodozyme® in 45 patients with 51 chronic wounds following the same methodology without differentiating infection status of the wound (Wood et al., 2010). Data from 30 wound care locations across England showed complete healing in 12%, improvement in 72%, no change in 14%, and deterioration in 2%, with an overall mean reduction in wound area of 20%. The most pronounced reduction in wound area was observed in surgical wounds (46%). Clinicians rated the dressing in 87% as better or much better than standard dressings (Wood et al., 2010). Although the mean wound area reduction was lower compared to the Oxyzyme® study, overall outcomes appeared preferable with 84% of wounds improving or healing completely compared to 73% in the Oxyzyme® study. However, no statistical analyses were performed. Although this mechanism was not directly evaluated, the authors suggest that the increased iodine concentration may not only exert an antimicrobial effect but also reduce inflammation. An additional case series by Lafferty et al. (Lafferty et al., 2011) investigated 11 patients with chronic wounds of mixed etiology applying Iodozyme® to infected wounds and Oxyzyme® to non-infected wounds. While the presented effects in wound healing are comparable to the previously mentioned case series, this study provided additional cost analyses for the 8 patients that completed 20 weeks of treatment to evaluate whether the increased cost of the dressing could be compensated by improved healing rates. With an average treatment cost of £85.40 per week per wound, Oxyzyme®/Iodozyme® treatment was approximately 11% more expensive than standard treatment with £76.80 per week per wound. However, according to the presented results with a complete healing rate of 60% over 20 weeks, 52-week total costs were calculated and compared to total costs under standard treatment with a healing rate of 8% over 24 weeks observed at the same center. This resulted in total savings of £23,036 for the cohort over 52 weeks, assuming the observed healing rate was consistent (Lafferty et al., 2011). Despite indicating a potential benefit, these calculations are subject to several limitations, including study design, the lack of a control group, and reliance on multiple assumptions, which reduce clinical validity. The highest level of evidence is provided by Moffatt et al. (Moffatt et al., 2014) in an RCT investigating the application of Oxyzyme® in venous and mixed venous-arterial ulcers compared to standard wound dressing. A total of 100 patients were recruited, all of which received concurrent compression therapy. Out of these, 47 were allocated to the experimental arm receiving Oxyzyme® or Iodozyme®, without defining indications for specific dressing choice and regular interchange between the two being mentioned. The remaining 53 patients were allocated to the control arm and received standard wound care, i.e., continuation of their previous treatment regimen. All patients were seen weekly, with additional dressing changes performed as needed. Wound assessment was performed after 12 weeks of treatment and repeated after 24 weeks. No statistically significant improvements in wound healing rates, quality of life, or pain reduction were observed in the experimental group. However, patients in the experimental arm required an average of 10 dressing changes over 12 weeks compared to an average of 15 for patients in the control arm, potentially improving cost-effectiveness for the experimental arm (Moffatt et al., 2014). Thus, high-level evidence failed to corroborate the positive effects on wound healing reported in earlier case series.

A more recent variation of peroxide dressings employs a different design, using a polymer containing sodium percarbonate and calcium peroxide as oxygen sources. This polymer is enclosed by an outer patch containing a hydrated gel layer required for the chemical reaction and a sealing polyurethane adhesive film. The inner layer consists of an alginate patch in contact with the oxygen-releasing polymer and a release paper ensuring diffusion into the wound. Tong et al. (Tong et al., 2026) performed a phase III randomized clinical trial evaluating this dressing for the healing of split-thickness skin graft donor sites in patients with burns of less than 30% total body surface area. The treatment group consisting of 68 patients received the oxygen-generating dressing and was compared to 69 patients receiving conventional petroleum jelly gauze dressings. All patients were followed for 21 days, with assessment of complete wound closure, wound healing rate, inflammation, infection, and pain. The oxygen-generating alginate dressing group exhibited faster wound healing rates during the first two postoperative weeks, after which the differences were no longer significant. However, time to complete re-epithelialization was significantly reduced in this group at 11 days compared with 15 days in the control group. Although the incidence of pain did not differ between the groups, the group receiving the oxygen-generating alginate dressing demonstrated significantly lower pain intensity scores than the control group. Moreover, inflammation and infection did not differ between the two groups (Tong et al., 2026). Despite these promising outcomes, the oxygen-generating alginate dressing required more frequent dressing changes, increased the workload for care personnel, and was eventually associated with higher treatment costs, thus limiting clinical applicability in its current form.

Taken together, peroxide-based dressings represent a promising approach to topical oxygen delivery by generating and releasing oxygen directly at the wound site while simultaneously exerting an antimicrobial effect. Early enzyme-based systems, such as Oxyzyme® and Iodozyme®, showed promising outcomes in several case series. However, these findings were not supported by subsequent higher-level evidence, although some cost-reduction advantages were observed. With more recent developments employing solid peroxides as an oxygen source, clinical applications have expanded from chronic wounds to acute superficial wounds undergoing persistent ischemia, particularly in burn care. Initial data from a phase III clinical trial demonstrates accelerated healing and a reduced time to re-epithelialization in split-thickness skin graft donor sites. Nevertheless, these advantages are offset by practical limitations, most notably increased demands on healthcare personnel and higher associated costs. Ultimately, peroxide-based dressings represent a biologically plausible approach for local oxygen delivery. However, current evidence remains inconsistent and largely indication-specific. High-quality data are lacking and cost-effectiveness remains uncertain, underscoring the need for well-designed RCTs. Consequently, widespread clinical adoption cannot currently be justified.

## 6 Microalgae-based OCs

Microalgae offer the potential for sustained oxygen delivery through *in situ* illumination. Moreover, incorporating microalgae into a biocompatible scaffold capable of integrating with the surrounding tissue permits subsequent skin grafting without necessitating scaffold removal. Such photosynthetic scaffolds were first demonstrated to generate oxygen under light stimulation *in vitro* and were subsequently validated in full-thickness skin defect animal models (Hopfner et al., 2014; Schenck et al., 2015).

To evaluate clinical feasibility, Obaíd et al. (Obaíd et al., 2021) conducted a phase 1 clinical trial using porcine-derived decellularized dermal matrices (Integra®; Integra Life Science Corporation, Plainsboro, NJ, United States of America) seeded with C. reinhardtii that were implanted in 8 patients with full-thickness skin defects due to various causes, including trauma, pathological scarring, and tissue necrosis. After initial surgical debridement, the scaffolds were fixed to the defect and illuminated in short dark/light cycles over 7 days with split-thickness skin grafting performed after adequate integration of the scaffold, usually after 21 days. Patients were then kept in hospital for an additional 6 days and follow-up was performed for at least 3 months. Successful integration of the scaffold and overlying skin grafts could be observed in all patients. Self-evaluation regarding pain, burning, itching, and smell yielded favorable results. Furthermore, blood samples collected throughout the follow-up period showed no abnormalities, other than a slight elevation in C-reactive protein in the first few days after surgery, most probably a consequence of the surgical trauma. Histological analyses of the adjacent tissue showed adequate integration of the scaffold without any overwhelming foreign body reaction as well as organized formation of neo-dermis on day 21 and successful integration of the skin graft on day 27. Microalgae showed an orderly structure and presence within the scaffold on day 7 and disappeared by days 21–27. Progressive neovascularization was observed in the adjacent dermis, and early blood vessels could be observed within the scaffold, however, without any vascular lumen. Thus, this study indicates successful scaffold integration and early vascularization (Obaíd et al., 2021). A longer 17-month follow-up was described in a case report following the original study, reporting the outcomes of a patient following correction of a contracted scar in the cubital fossa (Obaíd et al., 2022). At 17 months, the new tissue exhibited positive characteristics in hydration, trans-epidermal water loss, and flexibility, allowing for complete arm extension. Furthermore, favorable results in patient-reported outcomes for this patient could be shown using the SCAR-Q questionnaire (Klassen et al., 2018). However, this patient also received negative pressure wound therapy following skin graft surgery and further local vacuum therapy in five weekly sessions 16 months after graft surgery, potentially influencing the outcome (Obaíd et al., 2022).

In summary, microalgae-seeded scaffolds represent an innovative approach to local oxygen delivery, offering the unique capability of externally controlled oxygen generation. The available evidence is encouraging regarding safety and feasibility, with a key advantage being the combination of oxygen delivery and structural support. This may reduce the need for frequent dressing changes and facilitate the transition to definitive wound closure in full-thickness skin defects. However, evidence is currently restricted to a single phase 1 trial with heterogenous wound etiologies and a limited cohort size and a subsequent case report with additional, potentially confounding, treatments. Long-term outcomes, scalability, cost-effectiveness, comparison with current standard-of-care treatment, and potential applicability to other types of wounds remain to be explored in future studies.

## 7 Physical OCs

In an RCT conducted in a military setting, Lairet et al. (Lairet et al., 2014) investigated the application of OxyBand™ (OxyBand Technologies Inc., California, United States of America) on donor sites of split-thickness skin grafts. Oxyband™ is a dressing employing a central layer containing a gaseous oxygen reservoir enclosed by an occlusive outer layer and a high-transfer inner film allowing for continued oxygen diffusion. A total of 17 patients with burn wounds of less than 30% total body surface area requiring surgical management and at least two donor sites were included, allowing each patient to serve as their own control. After hemostasis, the donor sites were randomly assigned by means of a randomization table and treated with either Oxyband™ or standard antimicrobial bismuth tribromophenate dressing. The donor sites were inspected daily for 4 days and then every other day until healing, defined as ≥90% re-epithelialization, was achieved. They observed significantly faster healing times of 9 days in the donor sites treated with Oxyband™ compared to 12 days in control sites. Furthermore, early pain scores were significantly lower at the donor sites treated with Oxyband™ than at the control sites. Aesthetic outcomes were assessed between days 30–45 and did not show significant differences between the groups (Lairet et al., 2014).

A different wound dressing, which was later discontinued, employed a similar principle by encapsulating oxygen within a closed-cell foam hydrogel. Its effects on scar quality after abdominoplasty were assessed in a clinical trial without producing a final publication (Halyard Health, 2017). A small cohort of 8 patients with 4 wounds per patient was enrolled. The left and right side of the patient served as intraindividual case and control wounds and were randomized prior to treatment. Re-epithelialization and pain level were assessed 14 days after surgery and did not show any significant differences between the groups. Furthermore, scar quality was assessed at 42 days post-op, with no significant differences observed (Halyard Health, 2017). To date, no further clinical studies of this dressing have been reported.

Taken together, these findings suggest that physical OCs may represent a simple method of local oxygen delivery in selected patient groups. However, the very limited number of clinical studies with varying patient groups and mixed outcomes warrants further investigation before widespread clinical implementation could be considered.

## 8 Future directions

Promising avenues for the use of OCs in plastic surgery include multienzyme-mediated peroxide systems, nanoparticle-based carriers and novel types of OCs, such as perfluorocarbons (PFCs).

Enzyme-based systems often use enzymes with a catalase-like activity. Catalase converts hydrogen peroxide into oxygen and water without generating reactive oxygen species, thereby oxygenating the surrounding tissue and acting as an antioxidant (Steg et al., 2015). These systems may be particularly useful in chronic or infected wounds, such as diabetic foot ulcers. However, the availability of hydrogen peroxide within these types of wounds is limited, thus reducing the oxygenating effect of exogenous catalase only. The addition of peroxides within polymer matrices or nanoparticles to enable controlled release (Steg et al., 2015; Guan et al., 2021), or the incorporation of multi-enzyme cascades represent promising approaches. Preclinical studies of such methods report positive outcomes in wound healing applications (Wang et al., 2022), however, clinical studies have not yet been reported. A further approach involves the direct encapsulation of oxygen within a nanoparticle, commonly known as “nanobubbles”. These systems have demonstrated encouraging effects on wound healing in preclinical studies and may enable additional modifications, such as integrating exosomes into the “nanobubble” capsule (Han et al., 2024; Ren et al., 2023).

PFCs represent a novel type of OC-based on fluorinated carbon chains capable of binding and releasing nonpolar gases, such as oxygen and carbon dioxide, according to the partial pressure gradients (Wijekoon et al., 2013). However, PFCs are hydrophobic and, thus, cannot be dissolved in water, limiting their clinical applicability. To circumvent this, PFCs have been encapsulated in nanoparticles, conjugated to polymers or other nanomaterial surfaces, and incorporated in hydrogels to enable cellular oxygen delivery (Wijekoon et al., 2013; Wrobeln et al., 2017; Patil et al., 2016; Kumar and Kehr, 2022; Jalani et al., 2017; Yang et al., 2022). PFCs have been clinically employed as blood substitutes, although their FDA approval was later revoked due to unforeseen side effects (Jägers et al., 2021). Modern variations are currently under investigation for human use in myocardial infarction and stroke (Jägers et al., 2021), but their application in wound healing currently remains preclinical. Despite the fact that none of these emerging OCs have yet established clinical efficacy in wound healing, several preclinical studies have demonstrated encouraging results. The future clinical translation of these methods will depend on demonstrating safety, controlled oxygen release, scalability, and practical superiority over existing systems.

## 9 Conclusion

OCs represent an encouraging strategy based on fundamental biological principles to address persistent tissue hypoxia in a variety of situations encountered in plastic, reconstructive, and aesthetic surgery. By enabling oxygen delivery independent of vascular perfusion, they may complement conventional therapies for the management of acute and chronic wounds. Hemoglobin-based OCs, particularly Granulox®, have the most extensive clinical evidence in chronic wound healing. The discussed M101-based product HEMHealing® is comparatively less established with evidence currently limited to case reports in the treatment of burn wounds and flap necrosis. However, its markedly elevated oxygen-carrying capacity positions it as the most promising candidate for further clinical development within this category. Peroxide-based systems provide an alternative method of local oxygen delivery through *in situ* oxygen generation with additional antimicrobial properties. Despite their mechanistic appeal, the highest-quality evidence failed to demonstrate a benefit over standard care, and clinical adoption of early enzyme-based systems has been limited accordingly. More recent formulations, however, have shown promising results for split-thickness skin graft donor-site wounds, suggesting a potentially improved suitability for acute, time-limited applications rather than for chronic wound management. Recently, microalgae-seeded scaffolds have introduced the unique ability to externally control oxygen production while providing structural tissue support, with early studies supporting feasibility and safety. Physical OCs offer a mechanistically simple approach through sustained oxygen diffusion and have shown potential benefits in selected clinical applications, however, so far only in small trials and with mixed outcomes.

Despite these exciting developments, the overall clinical evidence for the efficacy of OCs remains limited by small patient cohorts, heterogeneous methodologies, and retrospective designs. High-level evidence could not consistently support the positive results observed in retrospective studies of chronic wound treatment. Consequently, definitive conclusions regarding the comparative efficacy and optimal indications of OCs in chronic wounds cannot yet be drawn. Nonetheless, the application of OCs as an adjunct to standard treatment in selected patient groups may provide clinically relevant improvements. Conversely, preliminary studies in acute wound settings, such as burn wounds, skin graft donor sites, and full-thickness skin defects, have generally reported positive outcomes, albeit large RCTs are still lacking to confirm these results.

Overall, the heterogeneity of evidence did not allow for a quantitative evaluation, such as a meta-analysis. However, to facilitate comparison and clinical interpretation, the products mentioned in this review article and their corresponding indications, reported adverse events, and commercial maturity are presented in Table 3. Furthermore, the authors give a recommendation for their clinical implementation specific to each indication based on the presented literature (Table 3).

**TABLE 3:** Comparison of different oxygen carrier products, as included in the present review.

Category	Product	Indication	Reported adverse events	Commercial maturity	Recommendation
Hemoglobin-based	Granulox®	Foot ulcers	None related to treatment	Commercialized	Insufficient evidence for routine use with inconsistent findings in RCT; may be considered for adjunctive use
		Other chronic wounds	None related to treatment	Commercialized	Conditional recommendation for adjunctive use in selected patients
		Burns	None reported	Commercialized, off-label	May be considered as adjunctive therapy in pediatric patients with <30% TBSA
	HEMHealing®	Burns	None reported	Commercialized	Insufficient evidence for routine use; may be considered as adjunctive therapy
		Skin flap necrosis	None reported	Commercialized	Insufficient evidence for routine use; may be considered as adjunctive therapy
Peroxide-based	Oxyzyme®/Iodozyme®	Chronic wound	Might cause inflammatory reaction and encourage autolytic debridement with increased exudate and pain	Commercialized	Insufficient evidence for routine use; may be considered in selected patients with the risk of increased discomfort/pain
	Polymer oxygenated alginate dressing	Skin graft donor site	None related to treatment	Investigational	Conditional recommendation for adjunctive use though not currently commercially available
Microalgae-based	Integra® + C. reinhardtii	Full-thickness skin defect	None reported	Investigational	Insufficient evidence for routine use; evidence suggests feasibility and safety though not currently commercially available
Physical	OxyBand™	Skin graft donor site	None related to treatment	Commercialized	Conditional recommendation for adjunctive use in selected patients
	OxyGenesys™	Scar formation	None related to treatment	Discontinued	No recommendation

RCT, randomized controlled trial; TBSA, total body surface area.

Future advances in biomaterials, nanotechnology, and bioengineering may expand the therapeutic potential of OCs. Emerging strategies, such as enzyme-based systems and PFC-based formulations, may allow OCs to overcome current limitations and broaden clinical applicability. To facilitate translation into clinical practice, future research should focus on larger studies with standardized outcome measures, long-term assessments, and direct comparisons with established treatment modalities. Overall, OCs represent an evolving field with growing clinical interest and the potential to improve patient outcomes in plastic, reconstructive, and aesthetic surgery by addressing one of the fundamental challenges of tissue repair: providing a breath of fresh air by adequate oxygenation.
